# The Epidemiological Profile of Open Globe Injuries and Prognostic Factors in a Tertiary Care Centre

**DOI:** 10.7759/cureus.15846

**Published:** 2021-06-22

**Authors:** Hui Ruan Ng, Shew Fei Chee, Khai-Siang Chai, Mei Fong Chong, Mushawiahti Mustapha

**Affiliations:** 1 Ophthalmology, Hospital Raja Permaisuri Bainun, Ipoh, MYS; 2 Ophthalmology, Pusat Perubatan Universiti Kebangsaan Malaysia, Kuala Lumpur, MYS; 3 Ophthalmology, Pusat Perubatan Universiti Kebangsaan Malaysia, Kuala lumpur, MYS

**Keywords:** open globe injuries, penetrating ocular injuries, epidemiology, prognostic factor, visual outcome

## Abstract

Purpose

To describe the epidemiology of open globe injuries and its prognostic factors from the perspectives of a tertiary care centre in northern Malaysia.

Methods

A retrospective study of open globe injuries in a period of three years between June 2017 and May 2020. Patients presenting with open globe injuries were identified and recruited from hospital census. Case records were retrieved and analysed after recruitment.

Results

A total of 114 patients with 118 open globe injuries were included in the study. Four patients had bilateral eye involvement. Men were found to have seven and a half times higher rate of ocular injuries than women. The predominant age group of ocular trauma presentation was in younger adults between the age of 21 and 30 years old. Work-related injuries account for almost half of the globe injuries (48.7%) followed by motor vehicle accident-related, domestic accident-related, and others. The significant factors related to the visual outcome are presenting visual acuity (VA), presence of relative afferent pupillary defect (RAPD) and vitreous loss.

Conclusion

The factors related to visual outcomes in this study may aid the clinician in determining the visual prognosis of ocular injuries. Young working males were the most susceptible group to sustain penetrating ocular injuries due to their job nature. Health education and safety at workplace are essential to reduce the occurrence of ocular injuries.

## Introduction

Ocular trauma is an important cause of preventable blindness worldwide. The World Health Organisation estimated that each year there is an estimated 55 million eye trauma cases that lead to restricted activities for more than one day a year, approximately 1.6 million cases of binocular blindness, 2.3 million people with bilateral low vision and almost 19 million with monocular blindness [1]. The large number of ocular trauma have placed great emphasis and challenge in the ophthalmology world on the successful repair of open globe injury and subsequent visual rehabilitation. Counselling and prognostication become an integral part of managing ocular trauma prior and after surgical repair of open globe injuries. The predictive factors for visual outcome after open globe injuries include pre-operative visual acuity (VA), relative afferent pupillary defect (RAPD), size and location of wound, uveal or vitreous prolapse, lens damage, hyphaema, presence of intraocular foreign body (IOFB) and number of surgeries done [2-5]. Based on extensive literature review, there is limited data on the epidemiology and outcome of open globe injuries in Malaysia. The latest study that was done in 2009 found that the most common cause of globe injuries were domestic accidents [6]. However, newer studies are required to help us better understand and keep updated with the current trend of globe injuries due to evolving changes. In this study, we aim to describe the epidemiology and risk factors affecting the final visual outcome of open globe injuries in our population.

## Materials and methods

This is a retrospective study from June 2017 to May 2020. We analysed the case records of patients who underwent surgical repair for open globe injuries within a period of three years at a tertiary referral eye care centre in Ipoh which is one of the main cities in the northern part of Malaysia. The centre receives a high volume of ophthalmic patients out of which a proportion of them presents with ocular trauma and open globe injuries. Cases were identified from the computerised admissions census. Cases with a minimum post-operative follow-up of three months, and those who had complete data about their initial and post-operative ophthalmologic examinations were included in the study. Permission to conduct the study was obtained from the local ethics committee. This research adhered to the Tenets of the Declaration of Helsinki.

Case records of 118 eyes from 114 patients with open globe injuries were reviewed. Classification for open globe injuries was based on the Birmingham Eye Trauma Terminology (BETT) [7]. The factors studied were demographic data, aetiology of injury, presenting visual acuity after injury, presence or absence of RAPD, mechanism of injury, and other associated ocular injuries as well as complications that arise from the injury.

For statistical analysis, the pre-operative VA and post-operative VA were grouped into two categories: ≤6/60 and >6/60. The relationship between different preoperative variables and the final VA was analysed by Statistical Package for the Social Sciences (SPSS) version 25.0 (IBM Corp., Armonk, NY) using correlation analysis for univariate analysis. Furthermore, binary logistic regression was performed for the purpose of multivariate analysis. The association between risk factors and final VA was considered statistically significant if P ≤ 0.05.

## Results

A total of 114 patients with 118 open globe injuries were included in the study. Four patients had bilateral eye involvement. Men (n = 104, 88.1%) were found to have seven and a half times higher rate of open globe injuries than women (n = 14, 11.9%). The predominant age group of ocular trauma presentation was in younger adults between the age of 21 and 30 years old. Most of the ocular injuries in this study has a propensity to involve the left eye (n = 70, 59.3%). Out of the 118 globe injuries, slightly more than half of the cases were of Malay ethnicity (n = 65, 55.1%), followed by Indians (n = 20, 16.9%), Chinese (n=16, 13.6%), and others (n=17, 14.4%) owing to the fact that the Malays constitute the largest ethnic group in Malaysia. The category of others involved foreigners from Indonesia, Bangladesh, and India. The majority of the patients (n=108, 91.6%) presented to the hospital within 24 hours of trauma. Unfortunately, there were five patients who presented one day later and another five presented two days after trauma. The mean follow-up duration for these patients were 4.7 (+/- 1.8) months. Approximately one-third of the cases had initial presenting VA of counting fingers or worse (n=45, 38.1%), 52 (44.1%) cases with VA of 6/24 to 6/60, 10 (8.5%) cases with VA of 6/12 to 6/18, and 11 (9.3%) cases with VA of 6/9 or better. At three months post-operative follow-up, the number of eyes with VA 6/9 or better improved to 22.9% (n = 27), 12 (10.1%) eyes had VA of 6/12 to 6/18, 29 (24.6%) eyes had VA of 6/24 to 6/60, and 50 (42.4%) eyes had VA of counting fingers and worse. Eighty-one (68.6%) eyes required a single surgery, while 34 (28.8%) eyes required two surgeries and the remaining three (2.5%) required three surgeries. 

**Figure 1:**
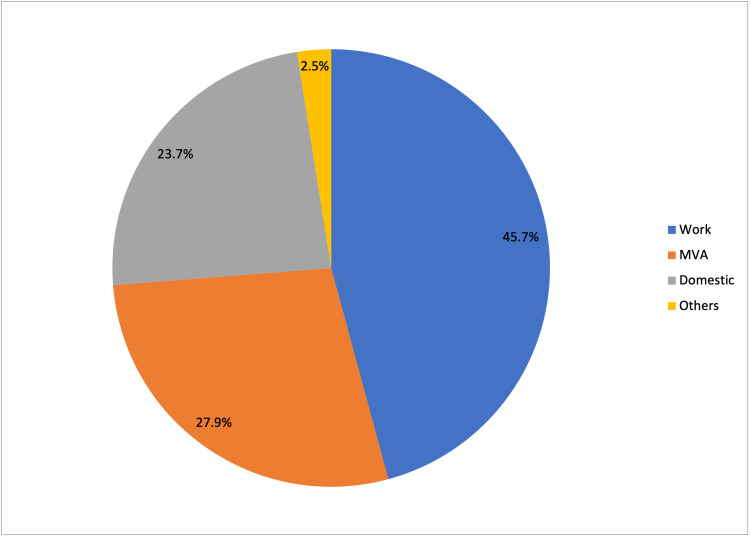
Distribution in percentages of penetrating ocular injuries (n = 118) by setting. MVA: motor vehicle accident.

**Figure 2:**
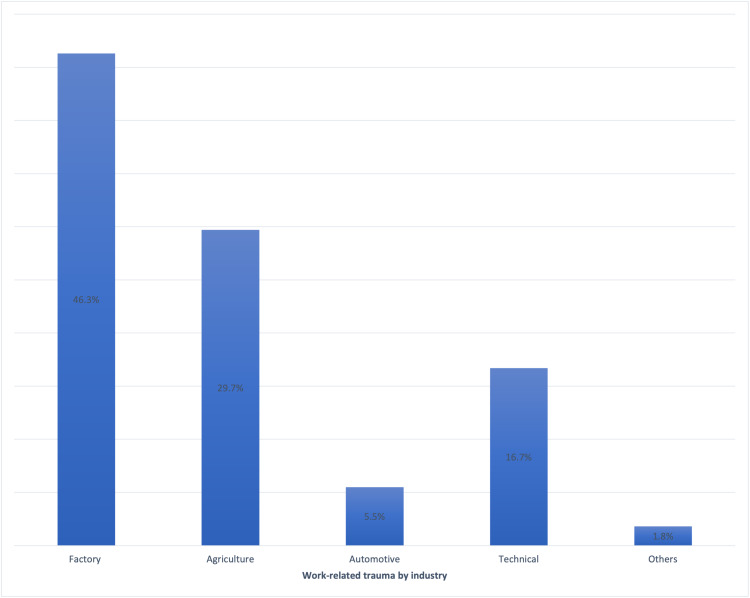
Distribution in percentages of work-related trauma (n = 54) by industry.

Work-related injury was the most common cause of penetrating ocular injuries, accounting for 54 (45.7%) cases, followed by motor vehicle accident-related (n = 33, 27.9%), domestic accident-related (n = 28, 23.7%), and others (n = 3, 2.5%) which includes sports-related and assault-related injuries (Figure 1). For work-related injuries, the factory was the most common setting for ocular trauma (Figure 2), followed by agriculture, automotive and technical. Among domestic accident-related injuries, half of them (n = 14, 50.0%) occurred while playing, followed by household activities (n = 7, 25.0%) and falls (n = 5, 17.9%), while the remainder were sustained in various ways. Playing activities include dealing with fire crackers during the festive seasons, handling toy guns and unsupervised usage of potentially sharp objects like clothes hanger. 

Lid laceration was an accompanying injury in 16 (13.6%) eyes. RAPD was present in almost half of the cases (n = 58, 49.1%). Hyphaema was noted in 42 (35.6%) eyes. Lens was found to be cataractous with or without anterior capsular rupture in 43 eyes (36.4%). Half of the eyes had vitreous loss (n = 62, 52.5%) while intraocular foreign body was seen in 27 (22.9%) eyes.

The preoperative variables that were statistically significant for predicting final visual outcome at three months post-operatively include initial presenting VA, presence of RAPD, eyelid laceration involvement, and vitreous loss based on the univariate logistic regression analysis (Table 1). All the significant variables in univariate analysis were added in the multivariate analysis using binary logistic regression to further analyse their associations with final vision outcome. Based on Table 2, the most statistically significant factors leading to poor visual outcome on multivariate logistic regression analysis are presenting VA, presence of RAPD and vitreous loss. 

**Table 1:** Univariate logistic regression: pre-operative variables affecting final VA with statistical calculations. RAPD: relative afferent papillary defect; IOFB: intraocular foreign body; CV: coefficient of variation; CI: confidence interval; VA: visual acuity.

	Odds ratio	P-value	CV	95% CI for CV
Initial VA	-2.726	0.000	0.065	0.024-0.177
RAPD	3.855	0.000	47.218	15.311-45.621
Eyelid involvement	1.366	0.029	4.333	1.165-16.125
Cataract	0.398	0.305	1.489	0.696-3.186
Hyphaema	0.961	0.018	2.613	1.181-5.785
Vitreous loss	3.182	0.000	24.091	9.166-63.321
IOFB	0.671	0.144	1.957	0.796-4.810

**Table 2:** Multivariate logistic regression: pre-operative variables affecting final VA with statistical calculations. RAPD: relative afferent pupillary defect; CV: coefficient of variation; CI: confidence interval; VA: visual acuity.

	Odds ratio	P-value	CV	95% CI for CV
Initial VA	2.045	0.004	0.129	0.032-0.528
RAPD	2.833	0.000	16.996	4.229-33.305
Eyelid involvement	-0.991	0.297	0.371	0.058-2.391
Vitreous loss	1.263	0.051	3.535	0.900-13.893

## Discussion

In most population-based studies, there is a strong tendency for open globe injuries to involve male [2-5,8-15]. Similarly in this study, male was the predominant gender. This reflects the predominant involvement of men in higher-risk activities namely work or sports-related activities and motor vehicle accidents. The majority of patients were in the younger age group (less than 40 years old) which is also similar to other studies till date [2-4].

In this study, poor presenting visual acuity is significantly associated with poor post-operative visual outcomes. This is in accordance with other studies that showed presenting VA is the most important prognostic factor in determining visual outcome [2-5]. Presence of RAPD and vitreous loss were statistically significant to predict post-operative visual outcomes in this study. The final visual outcome was worse if there was the presence of RAPD as seen in other studies [2-5]. Presence of vitreous loss was reported to be associated with poor visual outcome [8,13-15]. The postulation was due to the possible vitreoretinal traction which may potentially lead to retinal detachment [2-3]. 

Work-related eye injuries were the most common cause of penetrating eye injury, accounting for almost half of the cases. This is in accordance with other reported studies from both developed and developing nations [8,13-15]. In the past, domestic accident-related injuries were the most common cause of open globe injuries in Malaysia. The most common setting for work-related injuries in this study was in factories followed by agricultural and technical-related fields. As work-related injuries account for the highest proportion of penetrating ocular injuries, it is very important to highlight the need for the higher authorities to intervene and help to take preventive measures. This includes the education and enforcement of the usage of personal protective equipment (PPE) at workplace to prevent ocular injuries. Besides medical consequences of loss of vision, work-related eye injuries are associated with socioeconomic burden, which includes the cost of medical care, time off work, loss of income, and permanent disability. As patients’ hospital bills are heavily subsidised by the government in Malaysia, this also poses a financial burden to the government. Therefore, taking preventive measures such as reinforcing the law for mandatory PPE usage at workplace is one of the best approaches to be considered by the legislative council.

This research is however limited by the lack of data on the detail of personal protective equipment (PPE) usage during work, due to the retrospective nature of this study. A prospective study in the future would be able to gather more meaningful data.

## Conclusions

Factors that determine the visual outcome of penetrating ocular injuries include pre-operative VA, presence of RAPD and vitreous loss. These factors may aid the clinician in determining the prognosis of ocular injuries. Young working males were the most susceptible group to sustain penetrating ocular injuries due to their job nature. Promotion of health education and safety at workplace are therefore essential to reduce the occurrence of ocular injuries.
